# The role of EP-2 receptor expression in cervical intraepithelial neoplasia

**DOI:** 10.1007/s00418-020-01909-2

**Published:** 2020-08-26

**Authors:** Elisa Schmoeckel, Patricia Fraungruber, Christina Kuhn, Udo Jeschke, Sven Mahner, Theresa Maria Kolben, Thomas Kolben, Theresa Vilsmaier, Anna Hester, Helene Hildegard Heidegger

**Affiliations:** 1 Department of Pathology, LMU Munich, Thalkirchner Straße 36, 80337 Munich, Germany; 2 Department of Obstetrics and Gynaecology, University Hospital, LMU Munich, Maistrasse 11, 80337 Munich, Germany; 3 Department of Obstetrics and Gynaecology, LMU Munich, Marchioninistraße 15, 81377 Munich, Germany; 4 Department of Obstetrics and Gynaecology, University Hospital Augsburg, Stenglinstr. 2, 86156 Augsburg, Germany

**Keywords:** EP-receptor, EP2, Prostaglandin E2, CIN, Cervical intraepithelial neoplasia, Cervical cancer HPV

## Abstract

Prostaglandin induced signalling is involved in different cancers. As previously described, the EP3 receptor expression decreases with increasing stage of cervical intraepithelial lesions (CIN). In addition, in cervical cancer EP3 is an independent prognosticator for overall survival and correlates with FIGO stages. Currently the role of Prostaglandin 2 receptor 2 (EP2) in CIN is unknown. The aim of this study was to analyse the expression of EP2 for potential prognostic value for patients with cervical dysplasia. EP2 expression was analysed by immunohistochemistry in 33 patient samples (CIN1–3) using the immune-reactivity scoring system (IRS). Expression levels were correlated with clinical outcome to analyse prognostic relevance in patients with CIN2. Data analysis was performed using non parametric Kruskal–Wallis and Spearman rank sum test. Cytoplasmic expression levels of EP2 correlated significantly (*p* < 0.001) with different grades of cervical dysplasia. Median EP2-IRS in CIN1 was 2 (*n* = 8), 3 in CIN2 (*n* = 9) and 6 in CIN3 (*n* = 16). Comparing regressive (*n* = 3, median IRS = 2) to progressive (*n* = 6, median IRS = 4) CIN2 cases the median IRS differed significantly (*p* = 0.017). Staining intensity (*p* = 0.009) and IRS (*p* = 0.005) of EP2 and EP3 correlate inversely. EP2 expression level significantly increases with higher grade of CIN and could qualify as a potential prognostic marker for the regressive or progressive course in CIN2 lesions. These findings emphasize the significant role of PGE2 signalling in CIN and could help to identify targets for future therapies.

**Electronic supplementary material:**

The online version of this article (10.1007/s00418-020-01909-2) contains supplementary material, which is available to authorized users.

## Introduction

After breast-, colorectal- and lung cancer, cervical cancer represents the fourth most common malignant tumour in women worldwide (Wallis 2014; Watson et al. 2014). Approximately 500,000 women worldwide are newly diagnosed with cervical cancer per year. 260,000 women die from the disease each year (Gottlieb 2016; Jiang et al. 2015; Landy et al. 2016). Incidence and mortality of cervical cancer correlates negatively with the Human Development Index and varies extremely in geographic contexts (Wentzensen 2016). Regarding Germany, 4500 women were diagnosed with cervical cancer in 2014 and 1500 of these patients died tumour associated (Zentrum fur Krebregisterdaten 2019). After the implementation of Pap smear screening, which detects precursor lesions of cervical epithelium, incidence dropped considerably (Hester et al. 2019). The persistent infection with specific types of high-risk papillomaviruses is considered the main risk of intraepithelial neoplasia and especially in the development of cervical cancer (Schiffman et al. 2011). The precursor lesions were formerly called cervical intraepithelial neoplasia (CIN) and ranged from CIN1 to CIN3 (Santesso et al. 2016). In 2014 the histological WHO classification has been altered, and cervical intraepithelial neoplasia is referred to as squamous intraepithelial lesion (SIL) since 2014 (Lu and Chen 2014). The lesions are divided in low grade (LSIL) and high grade squamous intraepithelial lesions (HSIL). CIN2 and CIN3 are now combined in HSIL (Lu and Chen 2014). However, pathologists still specify their diagnosis with CIN2/CIN3 due to the risk of progression to a cervical carcinoma that may differ between CIN2 and CIN3 (Luo et al. 2018; Papoutsis et al. 2017). Consequently, the therapy options also vary from conservative approaches to surgical treatments (Saah-Briffaut et al. 2006). Young women in childbearing age could especially profit from a watchful waiting strategy as conization increases the appearance of pregnancy complications such as cervical insufficiency and preterm labour (Wilkinson et al. 2015). However, apart from the size of the lesion there is no established marker for the prediction of progression or remission of CIN2 lesions (Kühn et al. 2015).

Heidegger et al. previously indicated that the prostaglandin E2-receptor EP3 is an independent negative prognostic factor in cervical cancer patients. The expression levels and the clinical outcome were proven to correlate with tumour stage (Heidegger et al. 2017). In addition, Hester et al. demonstrated that EP3 receptor expression levels correlate inversely with grades of CIN (Hester et al. 2019). Our aim was to further investigate the role of prostaglandin receptors in cervical intraepithelial neoplasia. This study is focussed on the EP2 receptor, as it is unique among all EP receptors. The fact that it is not desensitized by Prostaglandin E2 (PGE2) sets it apart from other EP receptors and highlights its role in the deferred phases of cellular response (Kalinski 2012).

## Materials and methods

### Tissue samples

The cervical tissue samples used in this study were collected from patients treated between 2007 and 2014 in the Department of Gynaecology and Obstetrics from Ludwig-Maximilians-University of Munich, Germany. This cohort was analysed in previous studies from our group (Hester et al. 2019; Kolben et al. 2016; Vogelsang et al. 2020). Due to multiple sections the CIN lesions got lost on the slides in many cases, which therefor had been excluded from the present study.

In total, 38 tissue samples of cervical dysplasia were immunohistochemically stained with anti-EP2-antibody; the staining was successful in 33 cases. Of these, 8 were classified as CIN1, 9 as CIN2 and 16 as CIN3. On their first visit all patients were tested positive for high risk Human Papillomavirus (Hybrid Capture 2, Quiagen). Histopathological grade of dysplasia and diagnosis were confirmed by a second gynaecological pathologist. Regarding the CIN2 collective, only cases with either a histologically confirmed progress (*n* = 6) or regress (*n* = 3) were used. The follow-up interval for patients with CIN2 ranged from 5 to 14 months. The cases that were classified as CIN2 at the latest possible date and had been ranked as CIN3 previously, were defined as regress. CIN2, which had progressed from a former CIN1 were also defined as progress.

The tissue samples were eligible for this study after all routine histopathological diagnostic procedures were completed. The data of the patients were completely pseudonymized. All analytic procedures complied with the Helsinki Declaration guidelines (Reference No. 167-14). Informed consent of the patients was guaranteed before study participation. The Ethics Committee of the Ludwig-Maximilians-University (Munich, Germany) accepted the design of the study.

### Immunohistochemistry

**Fig. 1:**
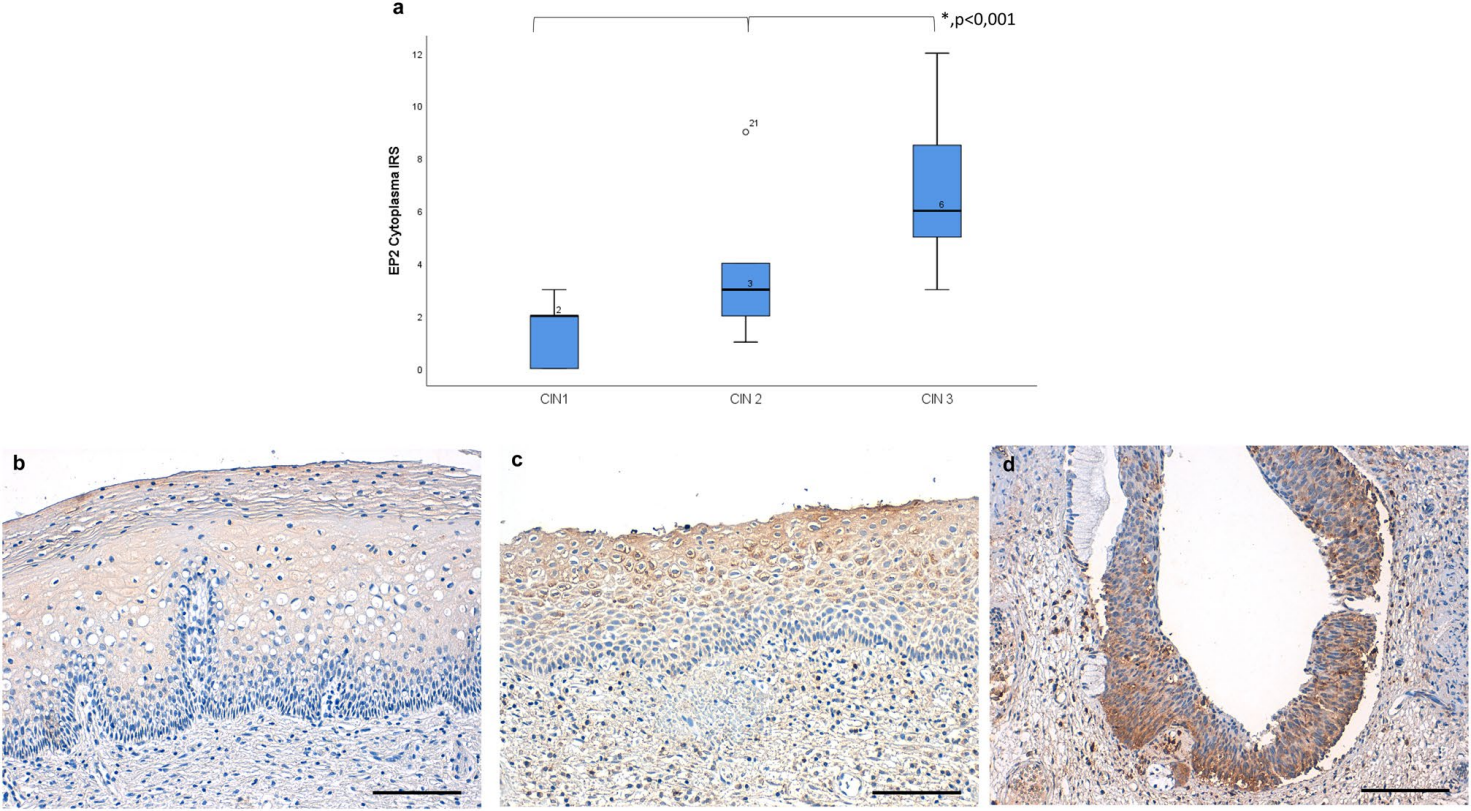
The expression of EP2-receptors in the cytoplasm increased significantly with increasing grade of cervical dysplasia, displayed by boxplots. The median value is stated above the median-line within the boxes (**a**). The images show representative microphotographs of EP2 staining in CIN1 (**b** IRS 2), CIN2 (**c** IRS 6) and CIN3 (**d** IRS 9). 200× magnification was used for picture b, c and d. Scale bars refer to 100 µm. Asterisk represents statistically significant differences in the staining results of CIN1-3

**Fig. 2:**
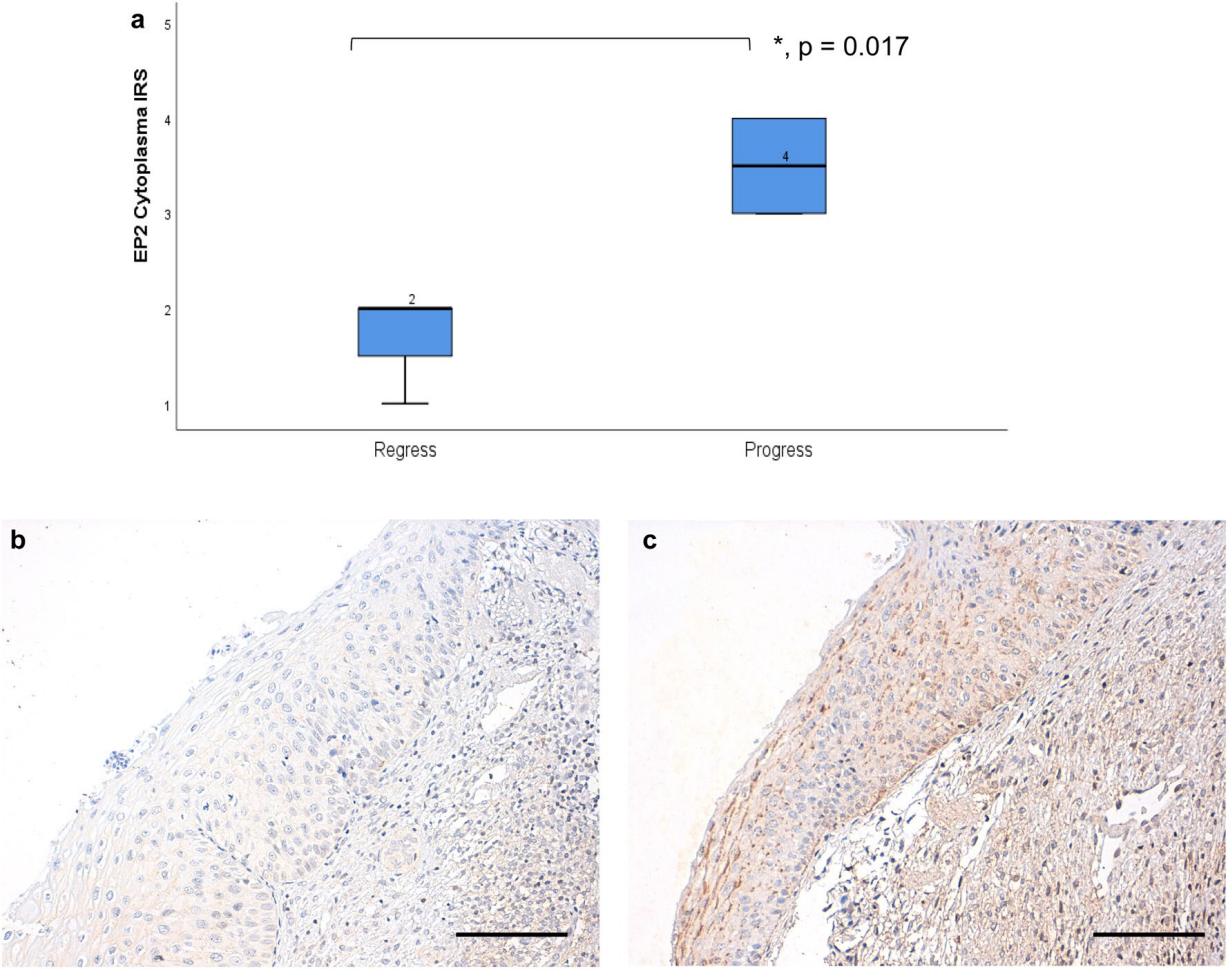
The median IR-score in regressive cases and in progressive cases differs significantly as shown by the boxplots (**a**). The different staining results with the anti-EP2-antibody in regressive (**b** IRS 1) versus progressive (**c** IRS 6) CIN2 samples (*p* = 0.017). 200× magnification was used for picture **b** and **c**. Scale bars refer to 100 µm. Asterisk represents statistically significant differences comparing regressive and progressive CIN2 cases

The immunohistochemistry of the paraffin-embedded cervical tissue samples was conducted as according to our IHC-protocol, which is provided in the supplement. First the samples were dewaxed for 20 min in xylol, then washed in 100% ethanol. In order to suppress the activity of the endogenous peroxidase slides were placed into 3% methanol/H_2_O_2_ for 20 min. Rehydration in a descending alcohol series followed. The slides were boiled in an airtight pot for 5 min at + 100 °C in a trisodium citrate buffer solution (Merck 244 and Merck 6448) with pH = 6 to unmask the antigen from formalin-fixation-associated protein-agglomeration. Washing in distilled water and PBS-buffer followed. The first diluent of the Polymer kit (ZytoChem Plus HRP Polymer System, Berlin, Germany) was applied for 5 min. The samples were incubated overnight at + 4 °C for 16 h with the anti-EP2-primary-antibody (anti-PTGER2 antibody polyclonal rabbit IgG; ABCAM, Cambridge, UK). After washing in PBS-buffer Reagents 2 (Post block) and 3 (horseradish peroxidase -Polymer) of the Polymer kit were administered. Substrate-staining was performed for two and a half minutes with DAB (chromogen substrate kit, Dako, Hamburg, Germany). Counterstain by hemalaun colouring and dehydrogenation in an ascending alcohol series followed, before the slides were mounted with “Eukitt” (Orsatec, Bobingen, Germany) (Heidegger et al. 2017). According to information in the human protein atlas sigma and placenta tissue were chosen as positive control. To get a negative control IHC staining was performed as characterized above, replacing the anti-EP2-primary-antibody by a rabbit negative control serum. The immune-reactivity scoring system (IRS, Remmele score) was applied to rate immunostaining semi-quantitatively using a Leitz (Wetzlar, Germany) microscope type Diaplan. The PL Fluotar objective lens provides a magnification/N.A 10/0.30. Images were captured with a JVC camera type KY-F55B with 440,000 pixel for PAL (JVCKENWOOD GmbH, Bad Vilbel, Germany) and the DISKUS acquisition software version 4.60.2017—#223 (Technisches Büro Hilgers, Königswinter, Germany). All pictures in this paper have got an image bit depth of 8bit in RGB-colourmodell. Figures 1d and 2c have got 150 dpi in height and width, all others have got 72 dpi. The IRS multiplies the intensity of the staining (0 = no, 1 = weak, 2 = moderate, 3 = strong staining) with the percentage of positive cells (0 = no staining, 1 ≤ 10% positive cells, 2 = 11–50% positive cells, 3 = 51–80% positive cells, 4 ≥ 81% positive cells). At an IRS of 0–1 the staining is negative, from 2–3 it is mildly positive, 4–8 is moderately positive and 9–12 strongly positive (Remmele et al. 1986). In order to obtain more precise numbers also the percentages were multiplied with the intensity. The analysis concerning the quantity of EP2-receptor expression in CIN2 was blinded for regress vs. progress of the dysplasia.

Immunohistochemistry regarding the EP3 were derived from a previous study performed by our group (Hester et al. 2019).

### Statistical analysis

For statistical analysis SPSS 25 (PASW Statistic, SPSS Inc., IBM, IL, USA) was used. To compare the expression of EP2 in varying levels of the cervical dysplasia the non-parametric Kruskal–Wallis rank-sum test was applied. The correlation between levels of EP3 and EP2 was tested with the non-parametric Spearman rank correlation test. *p* values ≤ 0.05 were considered as statistically significant. Figures were configured with SPSS 25 and Microsoft Power Point 2016 (Microsoft, Redmond, WA, USA).

## Results

### EP2 expression increases with progressing grade of cervical dysplasia

We compared the EP-2-IR-scores between the groups of CIN1-3 to analyse differences in EP2 expression levels. The expression of EP2-receptors in the cytoplasm increased significantly in correlation with increasing grade of cervical dysplasia as shown in Fig. 1a. This difference was statistically significant when each grade of dysplasia was compared to the next higher one. In CIN1 the median EP2-IRS in the cytoplasm was 2, in CIN2 incidents the value was 3 and in CIN3 cases the median EP2-IRS was 6 (*p* < 0.001).

Exemplary staining for all grades of CIN are shown in Fig. 1b–d.

### Cytoplasmic IRS of EP2 positive cells is higher in CIN 2 lesions with a progressive course of the dysplasia

To determine if EP2-receptor expression might serve as a prognostic factor in regard to a progressive or regressive course in cervical dysplasia, we compared EP2 expression between CIN2 cases with histologically confirmed regress or progress. Although the number of cases was little (*n* = 3 for regress, *n* = 6 for progress) the study revealed statistically significant differences between the cytoplasmatic IRS of EP2-receptor expressions. In regressive cases the median IR-score was 2, while it was 4 in progressive cases (*p* = 0.017) as shown in Fig. 2a. Figure 2b and c display the different staining in regressive (Fig. 2b) versus progressive (Fig. 2c) CIN2 samples (*p* = 0.017).

### Intensity and IR-Score of EP2 correlates negatively with EP3 IRS and intensity

**Fig. 3:**
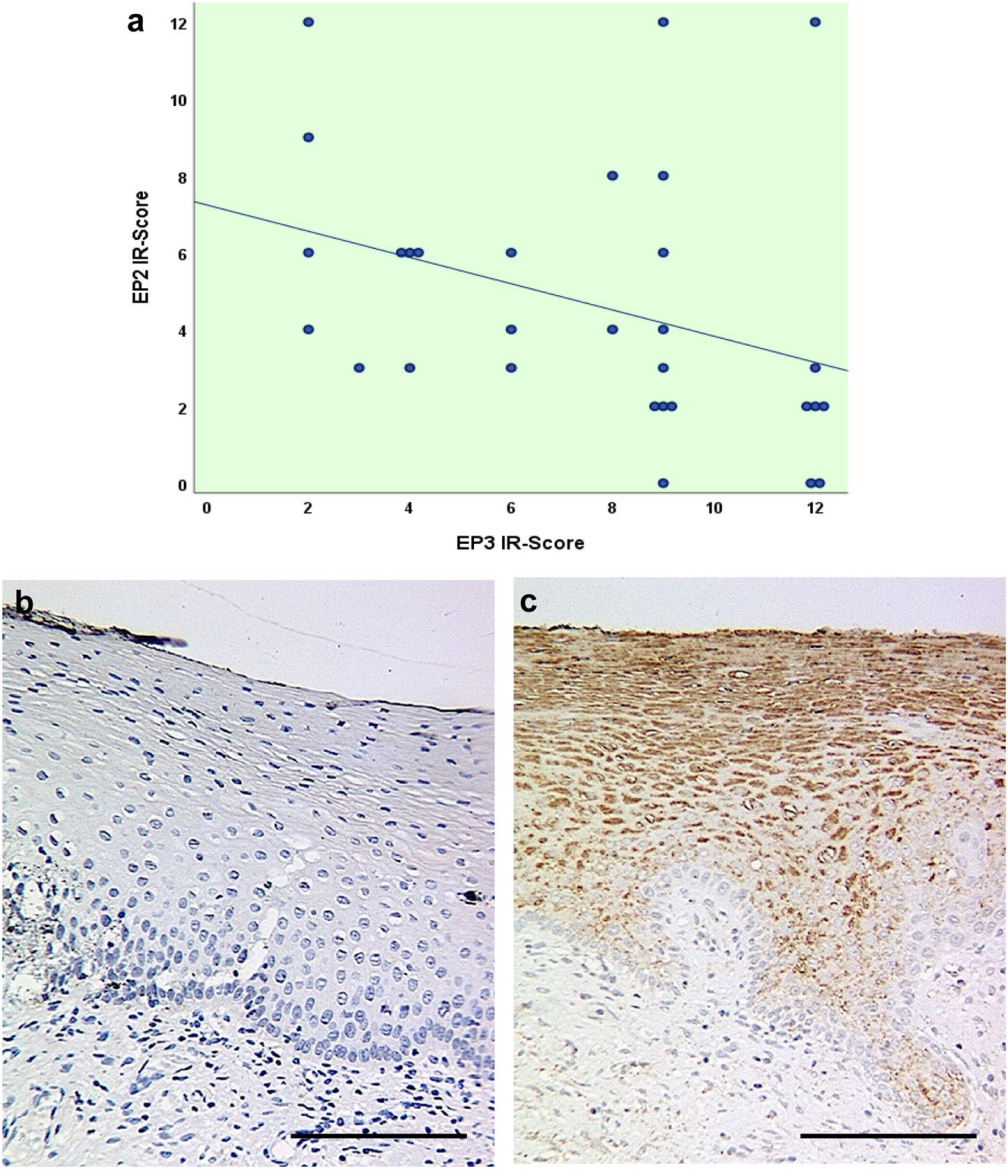
Correlation diagram for IR-score of EP2 and EP3 representing the inverse correlation of the two prostaglandin receptors in CIN tissues (**a**).The comparison of the staining results in a tissue sample of the same patient with CIN1 for a staining with the anti-EP2-antibody (**b**) and the anti-EP3-antibody (**c**) represents this inverse correlation. EP2 was not detected in the staining (IRS 0) whereas EP3 was seemingly highly expressed (IRS 12). 200× magnification was used for picture b and c. Scale bars refer to 100 µm

Former research indicated that EP3-receptor expression decreases with increasing grade of cervical dysplasia (Hester et al. 2019). The non-parametric Spearman correlation test revealed that both staining intensity and IRS of EP3 and EP2 correlate negatively on a significant level as presented in Table 1 and Fig. 3a.

**Table 1:** Correlation between EP3 and EP2 staining results

		EP3 intensity	EP3 IRS
Spearman-Rho	EP2 intensity		
	Correlation coefficient	− 0.470^**^	− 0.486^**^
	Sig. (2-tailed)	0.009	0.006
	*N*	30	30
	EP2 IRS		
	Correlation coefficient	− 0.465^**^	− 0.501^**^
	Sig. (2-tailed)	0.010	0.005
	* N*	30	30

^**^represent statistically significant differences at (*p* < 0.001)

*Sig*. significance, *N* number of cases, *IRS* immunoreactive score

Figure 3b and c show the comparison of the staining results in the same tissue sample of CIN1 for a staining with EP2 (IRS 0) and EP3 (IRS 12), representing the inverse correlation of the receptor types.

## Discussion

Herein we analysed the expression of the EP2 receptor in CIN samples for potential prognostic information for patients with cervical dysplasia. First, the level of EP2 receptor expression was compared to the grade of the dysplasia. In addition, we correlated the receptor expression to the clinical course of CIN2 samples. The analysis revealed that the median IR score of EP2 increases significantly with increasing grade of dysplasia (CIN1 = 2, CIN2 = 3, CIN3 = 6). CIN2 patients with a regressive clinical course had significantly lower EP2 levels compared to those with a progressive course. Therefore, increasing EP2 expression might indicate a progression of CIN towards cervical cancer.

The small number of CIN samples analysed (*n* = 33) is a critical limitation of our study. The study group proved to be adequate powered and well-reviewed by previous studies of our work group (Hester et al. 2019; Kolben et al. 2016; Vogelsang et al. 2020). However, due to several sections of the cervical biopsies, cases with missing CIN on the slide had to be excluded in the present study. The possibility of a colposcopy sampling error in the follow up check might represent an additional potential problem. In general, larger patient cohorts and further studies are needed to validate our findings.

The EP2 receptor is a G-protein coupled receptor with seven transmembrane domains bound to a heterotrimeric G-protein comprising the stimulatory Gαs and Gβγ subunits (Gilman 1987). It is physiologically activated by PGE2, a proinflammatory factor with immunosuppressive function (Phipps et al. 1991). PGE2 derives from arachidonic acids, which is firstly converted to prostaglandin H2 by cyclooxygenase 1/2 (COX 1/2) enzymes and further processed by PGE2 synthases (Lambeau and Lazdunski 1999). PGE2 is known to operate in many processes such as apoptosis, angiogenesis, chronic inflammation, tumour immunity, proliferation, migration and invasion (Kalinski 2012). Compared to other EP receptors, EP2 is interestingly not desensitized by PGE2 and therefore may contribute to deferred phases of cellular response (Nishigaki et al. 1996).

The Gα activation of the EP2 receptor can result in increased cAMP levels and activation of protein kinase A which regulates downstream transcription factors such as cAMP response element-binding protein (Fujino et al. 2005). Direct binding of Gα to regulator of G protein signalling promotes the release of glycogen synthase kinase-3β (GSK-3β) resulting in the activation of β-catenin pathway, which triggers the transcription of genes such as c-myc, cyclin d1 and vascular endothelial growth factor (Vaid et al. 2015). However, activation of serine/threonine-specific kinase (Akt) via Gβγ and phosphoinositide-3-kinase (PI3K) results in the inactivation of GSK-3β (Castellone et al. 2005). As a consequence, accumulated β-catenin can migrate to the nucleus to stimulate gene transcription via TCF/LEF family of transcription factors (Prasad and Katiyar 2014). When EP2 forms a complex with β-arrestin it can also function in a G protein-independent manner (Chun et al. 2009). With β-arrestin as a regulator EP2 can inaugurate pathways of PI3K, Akt, proto-oncogene tyrosine-protein kinase Src, extracellular signal-regulated kinases, c-Jun N-terminal kinases and epidermal growth factor receptors (Sun and Li 2018).

To this point, very little knowledge has been identified of the prostaglandin receptors in cervical intraepithelial neoplasia. Hester et al. showed that EP3 expression significantly decreases with higher grades of cervical intraepithelial neoplasia (Hester et al. 2019) and the expression levels of EP3 correlate with tumour stage as well as clinical outcome as Heidegger et al. could confirm (Heidegger et al. 2017). However, currently comparable studies analysing the expression of EP2 in cervical dysplasia are missing.

The role of EP2 has been studied in many malignancies as most of the induced pathways play a major role in cell proliferation, migration and angiogenesis (Bonanno et al. 2016; Sobhani et al. 2018). For instance, aberrant expression of EP2 has been found to be associated with chronic inflammation, deregulation of the immune system, angiogenesis, metastasis as well as multidrug resistance and has been observed in cancer of the colon, liver, breast and cervix (Asting et al. 2017; Cui et al. 2017; Gong et al. 2017; Huynh 2017). Besides the impact of EP2 activation on cell proliferation in cervical squamous intraepithelial lesions the immunosuppressive effect of EP2 seems of interest, as only HPV infections which are not cleared by the immune system can cause SILs and cervical cancer (Westrich et al. 2017).

HPV infections have to evade the host immune defence to persist (Schiffman et al. 2011). Incidence of HPV infections and HPV associated cancer is increased in patients with natural killer cell (NK) deficiencies (Orange 2013). Moreover, a strong cytotoxic T cell (CTL) response correlates with the regression of SILs (Woo et al. 2008). PGE2 contributes to an acute local inflammation. However, its prolonged immune response can shift cytotoxic T helper cell 1 (Th1), CTL and NK cell mediated type 1 immunity towards a Th2, Th17 and a regulatory T cell mediated immunity (Walker and Rotondo 2004). Thereby PGE2 prevents damage of lung or reproductive tissue (Huang et al. 2010; Vancheri et al. 2004). Although the limitation of type 1 immunity is pivotal for host self-preservation, it contributes to the establishment of infections with intracellular organisms and cancer, as they both depend on immunosuppression (Kalinski 2012).

den Boon et al. analysed the changes in gene expression patterns from HPV infected cervical tissue to cervical cancer. The study displayed that in early lesions, mostly genes functioning in DNA replication and cell division were upregulated. In transition from CIN3 to cancer the expression of genes serving the mitochondrial electron chain is reduced (den Boon et al. 2015). This suggests a switch from oxidative phosphorylation toward anaerobic glycolysis, and is known as the “Warburg effect” (Hsu and Sabatini 2008). As other DNA viruses, HPV sustain hypoxia inducible factor 1 alpha (HIF1α), possibly also endorsing the Warburg effect (Mazzon et al. 2013; Stover 2009). PGE2 also takes part in the induction of HIF1α (Jung et al. 2003).

Grabosch et al. revealed in a systemic review that non-steroidal anti-inflammatory drugs (NSAIDs) and selective COX2 inhibitors (celecoxib, rofecoxib) are not effective in the treatment of CIN (Grabosch et al. 2018). Other structures within the COX downstream signalling pathway like EP receptors might serve as alternative drug targets (Ganesh et al. 2018). Apart from that, levels of EP receptors such as EP2 and EP3 (Hester et al. 2019) might serve as potential prognostic biomarkers for patients with CIN2 lesions. In particular women in child bearing age, who might suffer from pregnancy complications after conization could benefit from additional prognostic information (Kühn et al. 2015).

## Electronic supplementary material

Below is the link to the electronic supplementary material.

Supplementary file1 (DOCX 12 kb)

## Data Availability

The datasets generated and analysed during the current study are available from the corresponding author on reasonable request.
