# Supplementary material for: The role of EP-2 receptor expression in cervical intraepithelial neoplasia
Source: Histochem Cell Biol. 2020 Aug 26;154(6):655–62. doi: 10.1007/s00418-020-01909-2 (PMC7723936; doi:10.1007/s00418-020-01909-2)
Supplement: Supplementary file 1 — Supplementary file1 (DOCX 12 kb) [file 418_2020_1909_MOESM1_ESM.docx]

**IHC – Protocol EP2 (PTGER2)**

Primary anti-body:

Anti-EP2; rabbit IgG polyclonal; concentration: 1000µg/ml

Company: Abcam, Cambridge, UK; Nr.: GR246965-1

Anti-EP3; rabbit IgG polyclonal ; concentration: 1000µg/ml

Company: Abcam, Cambridge, UK; Nr.:GR176558-2

Specifity: Basic Local Alignment Search Toolfinds analysis of the peptide immunogen showed no homology with other Human proteins.

Detection:

ZytoChem Plus HRP Polymer System (Mouse/Rabbit)

Company: Zytomed, Berlin, Germany; Nr. POLHRP-100

Chromogen-Substrat-Dilusion:

Liquid DAB+ Substrate Chromogen System

Company: Dako, Hamburg, Germany; Nr.: K3468

1) dewax the samples for 20 min in xylol

2) wash in 100% ethanol

3) place into 3% methanol/H2O2 for 20 min to suppress the endogenous peroxidase activity

4) rehydrate in a descending alcohol series

5) warm up the slides in a trisodium citrate buffer solution for 5 min at +100 ◦C (Merck 244/6448)

6) wash in distilled water and PBS-buffer

7) apply first diluent of the Polymer kit (ZytoChem, Berlin, Germany) for 5 min

8) incubate overnight at +4 ◦C for 16 h with the EP2 primary antibody OR EP3 primary antibody

(polyclonal rabbit IgG; ABCAM, Cambridge, UK)

9) wash 2x2 min washing in PBS

10) incubate for 20 min with Post Block (reagent 2)

11) wah 2x2 min in PBS

12) incubate for 20 min with horseradish peroxidase Polymer (reagent 3)

13) wash 2x2 min in PBS

14) develop with DAB (chromogen substrate kit, Dako, Munich, Germany) for two and a half minutes

15) counterstain for 2 min with hemalaun

16) dehydrogenate in an ascending alcohol series

17) mount with Eukitt (Orsatec, Bobingen, Germany)
